# Knowledge, attitude, and practice of artificial intelligence applications in medicine among physicians in Sudan: a national cross-sectional survey

**DOI:** 10.1097/MS9.0000000000002274

**Published:** 2024-06-17

**Authors:** Mohammed Hammad Jaber Amin, Gasm Alseed Abdelmonim Gasm Alseed Fadlalmoula, Musab Awadalla Mohamed Elhassan Elmahi, Noon hatim Khalid Alrabee, Lina Hemmeda, Mohammed Haydar Awad, Ghassan E. Mustafa Ahmed, Khabab Abbasher Hussien Mohamed Ahmed

**Affiliations:** a Faculty of Medicine, Alzaiem Alazhari University; b Faculty of Medicine, University of Khartoum, Khartoum; c Faculty of Medicine, Shendi University, Shendi; d Faculty of Medicine, Karary University, Omdurman, Sudan

**Keywords:** artificial intelligence, AI, KAP studies, physicians, Sudan

## Abstract

**Background and aims:**

Artificial intelligence (AI) has emerged as a rapidly developing tool within the medical landscape, globally aiding in diagnosis and healthcare management. However, its integration within healthcare systems remains varied across different regions. In Sudan, there exists a burgeoning interest in AI potential applications within medicine. This study aims to evaluate the knowledge, attitudes, and practices of AI applications in medicine among physicians in Sudan.

**Methods:**

The authors conducted a web-based survey cross-sectional analytical study using an online questionnaire-based survey regarding demographic details, knowledge, attitudes, and practice of AI distributing through various e-mail listings and social media platforms. A sample of 825 Physicians including doctors in Sudan with different ranks and specialties were selected using the convenient non-probability sampling technique.

**Result:**

Out of 825 Physicians, 666 (80.7%) of Physicians have previous knowledge about AI. However, only a small number 123 (14.9%) were taught about AI during their time in medical school, even fewer, just 120 (14.5%) had AI-related lessons in their training program. Regarding attitude, 675 (81.8%) agree that AI is very important in medicine, almost the same number, 681 (82.6%) support the idea of teaching AI in medical schools. Practically, 535 (64.8%) of doctors, think that should get special training in using AI tools in healthcare. Excitingly 651 (78.9%) of physicians are interested in working with AI in future. Based on different ranks of doctors toward AI; Medical Officers exhibited the highest proportion at (32.7%) of knowledge and understanding of AI concepts, followed by House Officers at (16.7%) (*p*=0.076); regarding attitude, Medical Officers demonstrated the highest (31.6%) favorable attitude, followed by House Officers at (17.5%) (*p*=0.229); In practice also, Medical Officer showed the highest portion (28.0%) among participants (*p*=0.129).

**Conclusion:**

While there is a positive attitude and some level of AI practice, there remains a considerable gap in knowledge that needs addressing.

## Introduction

Highlights

- Globally, artificial intelligence (AI) is becoming a more useful tool for diagnosis and healthcare management in the medical field. Nonetheless, there are regional variations in its adoption into healthcare systems. Potential medical uses of AI are attracting increasing interest in Sudan.
- The objective of this research is to assess Sudanese physicians’ knowledge, attitudes, and behaviors about AI applications in medicine.
- We sent an online questionnaire-based survey through many e-mail listings and social media platforms, utilizing a cross-sectional analytical study methodology to gather data on demographics, knowledge, attitudes, and AI practices. By employing the practical non-probability sampling approach, a sample of 825 physicians—including physicians in Sudan with varying levels and specialities—was chosen.

Artificial intelligence (AI) is transforming healthcare by using smart machines to perform tasks that usually need human thinking, as described by Marvin Minsky^1^. AI in medicine is growing rapidly due to the availability of powerful computers and big sets of data^2^. It is mostly helpful with diagnosis using technology like neural networks, which can find problems in X-rays, detect eye issues from pictures, identify skin cancer, and even guess genetic diseases from faces^3–7^. AI also helps manage ongoing health issues, makes decisions, finds new drugs, and works in surgery^8,9^. In healthcare, AI helps reduce mistakes in diagnoses^10,11^. Radiology, which deals with digital images, is a good fit for AI helping with diagnosing and finding problems in these images^12,13^. AI is also helping train new doctors^14^. But even with all these advantages, using AI in healthcare has tough challenges. Physicians might not want to change, there might not be enough finances, not enough trained people to set rules for using AI, not enough data on what people think, worries about replacing doctors, keeping secrets safe, and legal issues^14,15^. These problems show that putting AI into healthcare needs careful planning.

During ongoing armed conflicts in Sudan, healthcare facilities and workers have limited resources to provide healthcare to patients. Physicians only depend on telephones and the internet to diagnose and prescribe treatment for patients. Sudan finds itself in the nascent stages of introducing and achieving AI applications in medical fields, as there are limited studies about the application of AI in the medical field among physicians in Sudan, we decided to perform this study to evaluate the knowledge, attitude, and practice of AI applications in medicine among Sudanese doctors and help in the development of this field in Sudan. To our knowledge, this is the first study focusing on the KAP towards AI among Sudanese doctors.

## Materials and methods

### Study design

This study was a web-based survey cross-sectional analytical study conducted by physicians in Sudan using social media apps (WhatsApp, Facebook, and Messenger) and e-mail. Due to logistics issues in terms of cost and man-power, a convenience non-probability sampling technique was used to pick the sample population, during the period from the 1 October to the 3 October 2023. The bias that could exist from convenient sampling was bypassed via selecting a large sample size, thus reducing the risk of such bias.

### Study population

A total of 825 physicians in Sudan with different ranks and specialties were included in this study.

### Data collection and analysis

Data were collected carefully via a standardized pre-designed online survey on Google form questionnaires from physicians during the period of study. After that data were cleaned and checked for consistency before it entered for analysis. Finally, data were analyzed using the Statistical Package for Social Science (SPSS) version 22, and the results were expressed as tables in the result section.

### Measurements

#### Demographic information

The questionnaire consists of age, gender, and rank, for the Sudanese doctors.

#### Knowledge of AI

This sub-scale has seven questions about the knowledge of AI, including knowing about artificial intelligence, subtypes of AI like machine learning, applications of AI in the medical field, taught about AI in medical school, applications of AI in radiology and pathology, and AI during the training for post-graduate doctors (for the statistical analysis, yes = 1, & no = 0).

#### Attitude towards AI

This sub-scale has eight questions about the attitude toward AI, including the requirement of AI in the medical field, taught and training of AI in medical school and training programs, diagnosis and assessment of the disease, AI in pathology and radiology fields, replacing physicians in future, errors in diagnosis and burden of AI (for the statistical analysis, don’t know, disagree or strongly disagree = 0, & agree or strongly agree = 1).

#### Practice towards AI

This sub-scale has five questions about the practice of AI, including applied AI in any field, interest in using AI in future, and other questions (for the statistical analysis, yes = 1, & no, never applied = 0). The work has been reported in accordance with STROCSS guidelines^16^.

## Results

### General characteristics of study participants

A total of 825 participants were included in this study, age (mean 29.46 **±** SD 5.343), with a predominant age range of 20–30 years representing 576 (69.8%) of the sample. In terms of gender, males constitute the majority at 576 (69.8%) of the surveyed population. Professionally, medical officers hold the highest proportion at 342 (41.5%), trailed by house officers at 180 (21.8%). Registrars and specialists stand at 144 (17.5%) and 132 (16%) respectively, while consultants account for the smallest portion at 27 (3.3%). As shown in (Table 1).

**Table 1:** General characteristics of study participants.

Characteristic	value	Frequency	Percentage (%)
Age	20–30 years	576	69.8
	31–40 years	210	25.5
	41–50 years	36	4.4
	> 50 years	3	0.4
Sex	Male	576	69.8
	Female	249	30.2
Rank	House Officer	180	21.8
	Medical Officer	342	41.5
	Registrar	144	17.5
	Specialist	132	16
	Consultant	27	3.3

### Descriptive statistics for knowledge of AI in the medical field among participants

A lot of doctors, about 666 (80.7%) have heard and know about AI, but when it comes to understanding specific parts of AI, like Machine Learning and Deep Learning, only around 252 (30.5%) are familiar with them. About 318 (38.5%) of the doctors know that AI is used in medicine, but, surprisingly, only a small number, just 123 (14.9%) were taught about AI during their time in medical school. Even fewer, just 120 (14.5%), had any AI-related lessons in their training program. When it comes to AI being used in areas like Radiology and Pathology, the numbers are quite low at 219 (26.5%) and 141 (17.1%) respectively (Table 2).

**Table 2:** Descriptive statistics for knowledge of artificial intelligence.

Characteristic	Frequency	Percentage (%)
Do you know what artificial intelligence is?		
Yes	666	80.7
No	159	19.3
Do you know about machine learning and deep learning (subtypes of AI)?		
Yes	252	30.5
No	573	69.5
Do you know about any application of AI in the medical field?		
Yes	318	38.5
No	507	61.5
Have you ever been taught about artificial intelligence in medical school?		
Yes	123	14.9
No	702	85.1
Do you know about the application of AI in radiology?		
Yes	219	26.5
No	606	73.5
Do you know about the application of AI in the pathology field?		
Yes	141	17.1
No	684	82.9
Does your training include a curriculum regarding AI?		
Yes	120	14.5
No	705	85.5

AI, artificial intelligence.

### Descriptive statistics for attitude of AI in medical among participants

An overwhelming majority of Sudanese doctors, around 675 (81.8%) agree that AI is very important in medicine. Almost the same number, about 681 (82.6%) support the idea of teaching AI in medical schools. In healthcare practice, 645 (78.1%) believe that AI is really helpful for quickly finding diseases early and understanding how severe they are. However, there are worries too. A relatively portion of physicians 264 (32%), worry that AI will replace them in the future, also about 354 (42.9%) are concerned that AI might cause mistakes in diagnosing illnesses. Still, many doctors agree that AI is super important in specific medical areas like radiology 594 (72%) and pathology 561(68%) (for more details see Table 3).

**Table 3:** Descriptive statistics for attitude of artificial intelligence.

	Frequency/percentage, *n* (%)				
Characteristic	Strongly agree to	Agree	Don’t know	Disagree	Strongly disagree

Do you believe AI is essential in the medical field?					
	264 (32.0)	411 (49.8)	129 (15.6)	12 (1.5)	9 (1.1)
Do you think AI should be included in the curriculum in medical school as well as specialist training?					
	300 (36.4)	381 (46.2)	108 (13.1)	27 (3.3)	9 (1.1)
Do you think that AI aids practitioners in early diagnosis and assessment of the severity of disease?					
	219 (26.5)	426 (51.6)	138 (16.7)	30 (3.6)	12 (1.5)
Do you believe that AI will replace physicians in the future?					
	78 (9.5)	186 (22.5)	231 (28.0)	189 (22.9)	141 (17.1)
Do you believe AI is very essential in the field of radiology?					
	210 (25.5)	384 (46.5)	207 (25.1)	15 (1.8)	9 (1.1)
Do you believe AI is essential in the field of pathology?					
	189 (22.9)	372 (45.1)	222 (26.9)	36 (4.4)	6 (0.7)
Do you believe AI would be a burden for practitioners?					
	96 (11.6)	270 (32.7)	282 (34.2)	135 (16.4)	42 (5.1)
Do you believe AI would increase the percentage of errors in diagnosis?					
	105 (12.7)	249 (30.2)	291 (35.3)	144 (17.5)	36 (4.4)

AI, artificial intelligence.

### Descriptive statistics for practice of AI in the medical field among participants

Regarding the practice of AI in the medical field (Table 4), about 219 (26.5%) of physicians have used AI, but more than half 489 (59.3%) have not tried AI yet, and 117 (14.2%) never have. Some, around 297 (36%) found that AI makes their tasks easier, but more, about 423 (51.3%) of doctors did not feel this way. In healthcare, 660 (80%) of physicians think that doctors play a big role in using, understanding and evaluating AI technology in the medical field. Also, 535 (64.8%) of the doctors, think that should get special training in using AI tools in healthcare. Excitingly, 651(78.9%) of physicians are interested in working with AI in future (Table 4)

**Table 4:** Descriptive statistics for the practice of artificial intelligence.

Characteristic	Frequency	Percentage (%)
Have you ever applied AI technology in any field?		
Yes	219	26.5
No	489	59.3
Never applied	117	14.2
Did AI make your task easy?		
Yes	297	36.0
No	105	12.7
Never applied	423	51.3
Do you think physicians have a role important in the application and evaluation of AI technology in the medical field?		
Yes	660	80.0
No	27	3.3
Never applied	138	16.7
Would you like to work on AI in future?		
Yes	651	78.9
No	51	6.2
Never applied	123	14.9
Do you think that doctors should receive specific training on the use of AI tools in healthcare?		
Yes	535	64.8
No	155	18.8
Never applied	135	16.4

AI, artificial intelligence.

### Knowledge, attitude, and practice of AI based on the rank of the doctors

Knowledge across diverse positions showed that the majority of respondents (77.1%) reflect a poor understanding of AI concepts. Notably, Medical Officers exhibited the highest proportion at (32.7%), followed by House Officers at (16.7%), reporting poor understanding of AI concepts (*p*=0.076). Attitudinally, a dominant positive tendency toward AI technology was noted across (75.3%) majority of doctors, with Medical Officers demonstrating the highest (31.6%) favorable attitude, followed by House Officers at (17.5%) compared to other positions (*p*=0.229). Regarding the application and utilization of AI in practice, more than half of the participants (62.8%) had good practice of AI, with Medical Officers showing the highest portion (28.0%) among participants, followed by House Officers at (12.4%) compared to other participants (*p*=0.129) (Table 5).

**Table 5:** Knowledge, attitude, and practice of artificial intelligence based on the rank of the doctors

	*N* (%)		
Characteristic	Knowledge of AI	Attitude of AI	Practice of AI
	Good 189 (22.9)	Favorable 621 (75.3)	High 518 (62.8)
Rank	Poor 636 (77.1)	Unfavorable 204 (24.7)	Low 307 (37.2)
House Officer	42 (5.1)	144 (17.5)	102 (12.4)
	138 (16.7)	36 (4.4)	78 (9.5)
Medical Officer	72 (8.7)	261 (31.6)	231 (28.0)
	270 (32.7)	81 (9.8)	111 (13.5)
Registrar	30 (3.6)	102 (12.4)	91 (11.0)
	114 (13.8)	42 (5.1)	53 (6.4)
Specialist	33(4.0)	93 (11.3)	78 (9.5)
	99 (12.0)	39 (4.7)	54 (6.5)
Consultant	12 (1.5)	21 (2.5)	16 (1.9)
	15 (1.8)	6 (0.7)	11 (1.3)
*P*	0.076	0.229	0.129

Knowledge [(yes=1, no=0), (0–3=poor, 4–7=good), (total score=7)]. Attitude [(Don’t know, disagree or strongly disagree = 0, & agree or strongly agree = 1), (0–3=unfavorable, 4–8= favorable), total score 8]. Practice [(yes = 1, & no, never applied = 0) (0–2=low, 3–5=high), total score 5].

AI, artificial intelligence.

## Discussion

Artificial intelligence enhances clinical decision-making, streamlines hospital operations and management, improves medical image analysis, and transforms patient care and monitoring with the utilization of AI-driven wearable devices. In this online survey of 825 physicians in Sudan, we evaluated their knowledge, attitude, and practice toward applications of AI in the medical fields. Findings in this study exhibited that physicians in Sudan have a relatively high awareness of AI, with 666 (80.7%) of respondents claiming familiarity with the term, slightly lower than a similar study conducted among pediatricians in France (90%)^17^. However, when delving into the subtypes of AI like machine learning and deep learning, Sudanese doctors’ knowledge stands at 252 (30.5%), which again is slightly below to level of knowledge among pediatricians in France at (31%)^17^.

Compared with other cross-sectional studies conducted in developing countries like Syria and Pakistan doctors display percentages of (34.7%) and (35.3%), respectively^18,19^. Analysis revealed low-level knowledge about subtypes of AI 252 (30.5%). These discrepancies between the findings of our study and similar studies could be attributed to methodological variations in terms of sample size and sample selection. In addition, such findings give an insight into the depth of coping with updated knowledge and medical education in the study settings.

Regarding the application of AI in the medical field, our doctors report 318 (38.5%) awareness, surpassing Syrian (23.7%) and Pakistan’s (23.2%) doctors, with about 219 (26.5%) of our doctors claiming to know about the application of AI in radiology field which is higher than awareness of Syrian (21.2%) and Pakistan’s (24.7%) doctors, respectively^18,19^. In terms of the curriculum of AI in medical school and training programs, 123 (14.9%) of our participants reported being taught about AI in medical school and about 120 (14.5%) of them stated that their training program includes a curriculum regarding AI, which is slightly higher than another result of a study conducted by Ooi and colleagues among radiologists, which showed that only (5%) of respondents stated that they had received training in AI^20^. This highlights the need for incorporating AI education into medical school and training programs to enhance doctors’ understanding and ability to utilize AI tools effectively.

Regarding their attitudes toward AI in medicine, Sudanese doctors express a more reserved stance towards AI in medicine compared to their counterparts in Syria, the USA^21^, and Pakistan^22^. For instance, in Sudan, only 264 (32.0%) strongly agree that AI is essential in the medical field, whereas in Syria, a larger proportion (45.7%) strongly agreed with this notion^18^. This discrepancy indicates a more cautious embrace of AI significance in Sudanese medical practice, this can be explained by; the fear of Sudanese doctors from losing their jobs because of AI, as about 264 (32.0%) of them believe that AI will replace physicians in the future compare with low portion founded in both dermatology (5.4%)^23^ and physician of psychiatric (3.8%)^24^, and the refuse of Sudanese doctors to inclusion AI in medical school curricula, as only 300 (36.4%) of participants were strongly agreed, contrasting with higher percentages in Pakistan (83%)^19^. Moreover, regarding AI’s role in aiding practitioners in early diagnosis and disease severity assessment, Sudanese respondents again demonstrate more conservative opinions. While 219 (26.5%) strongly agree that AI aids in early diagnosis (compared to 37% in Syria)^18^, and 426 (51.6%) agree (compared to 45.8% in Syria)^18^, the proportion of strong remains notably lower in Sudan. However, instead of poor knowledge and attitudes toward AI in medicine among Sudanese doctors, most of the 651 (78.9%) show a high level of capability and interest in applying AI technology in clinical practice, and about 297 (36.0%) of them believe that AI tools will make the clinical practical life easier.

When we assessed the knowledge, Attitudes and Practices of Sudanese doctors concerning AI across all different ranks and functional levels of doctors, we found that a notable portion across all ranks perceives their knowledge of AI as poor with percentages varying from 12 to 33%, with Medical Officers have the highest poor knowledge at 270 (32.7%) followed by House Officers at 138 (16.7%). There is a generally positive attitude towards AI, with a higher percentage of doctors expressing a good attitude compared to those with a poor attitude, most of them were Medical Officers at 261 (31.6%) followed by Registrars at 102 (12.4%). However, the good attitude percentages remain below (32%) across all functional levels.

The practice of AI among Sudanese doctors appears moderate, with percentages indicating a higher proportion engaging in a good practice compared to those exhibiting a poor practice. Nevertheless, the good practice percentages range from 1.9 to 28%, with the majority of them being Medical Officers at 231 (28.0%), followed by House Officers at 102 (12.4%) indicating a varying level of integration of AI into their medical practices. Given the crucial need to support AI literacy among Sudanese doctors, we strongly recommend an urgent integration of comprehensive AI education within medical school curricula and ongoing training programs. They are simultaneously, fostering positive attitudes through awareness campaigns regarding AI’s role in enhancing, not replacing doctors and medical personnel. This proactive approach will prepare doctors to harness AI potential effectively, ensuring optimal patient care in Sudan’s evolving healthcare landscape.

This study contains limitations. There was a selection bias as we used a convenience non-probability sampling technique to collect the data, due to the ongoing war in Sudan it was difficult for us to estimate all the numbers or to access all Physicians in Sudan. Also, this study might not encompass the full spectrum of AI knowledge, attitudes, and practices among all Physicians in Sudan due to the selected timeframe, limited sample size, and potential variations in access to technology or differing levels of exposure to AI across regions or medical specialties, so we recommend performing more studies about AI among Sudanese doctors in the future. In addition, it is recommended that further studies adopt up-to-date methods in data handling and analysis, such as *“text mining”* to draw specifically related articles^25^, *“social network mining”* ^26^, and *“data mining”* for relevant data bases^27^.

## Conclusion

The data underscore the necessity for targeted educational initiatives to enhance understanding and familiarity with AI concepts among Sudanese doctors. While there is a positive attitude and some level of AI practice, there remains a considerable gap in knowledge that needs addressing. Bridging this gap through comprehensive training programs could significantly improve the integration and utilization of AI in their medical practices, ultimately enhancing patients’ care and outcomes.

## Ethical approval

Alzaiem Alazhari University’s ethics committee approved this study. Written informed consent was obtained from the local ethical committee and administration of Alzaiem Alazhari University. All protocols in this study were done according to the Declaration of Helsinki (1964).

## Consent

Written informed consent was obtained from the patients for publication and any accompanying images. A copy of the written consent is available for review by the Editor-in-Chief of this journal on request.

## Source of funding

The authors received no financial support for the research, authorship, and /or publication of this article.

## Author contribution

M.H.J.A. conceived the idea, wrote the manuscript and led this work. G.A.A.G., M.A.M.E., N.H.K.A., L.H., M.H.A., G.E.M.A. and K.A.H.M.A. did a write-up of the manuscript; and finally, K.A.H.M.A. reviewed and revised the manuscript for intellectual content critically. All authors reviewed, edited and approved the final version of the manuscript.

## Conflicts of interest disclosure

The authors declared no potential conflicts of interest concerning the research, authorship, and /or publication of this article.

## Research registration unique identifying number (UIN)

1. Name of the registry: Not required (because this was an observational cross-sectional study).
2. Unique Identifying number or registration ID: Not applicable (because this was an observational crosss-ectional study).
3. Hyperlink to your specific registration (must be publicly accessible and will be checked).

## Guarantor

Khabab Abbasher Hussien Mohamed Ahmed.

## Data availability statement

The data that support the findings of this survey are available on request from the corresponding author.

## Provenance and peer-review

Not commissioned, externally peer-reviewed.

## Collaborators List

Zuhal Yahya Mohamed Omer, Alzaiem Alazhari University, Faculty of Medicine, Khartoum, Sudan, E-mail: Zhlyhy01@gamil.com; Esra Mohammed Osman Meisara Seed Ahmed, Alzaiem Alazhari University, Faculty of Medicine, Khartoum, Sudan, E-mail: esramohammed99@gmail.com; Hadia Abdelrahman Hassan Osman, Sinnar University, Faculty of Medicine, Sinnar, Sudan, E-mail: hadoo.111@hotmail.com; Nada Abdalla Hassan Abdalkareem, Ahfad University for Women, Faculty of Medicine, Khartoum, Sudan, E-mail: Nadoya1998@outlook.com; Ammar Alemam Diab Alnour, Al Mughtaribeen University Faculty of Medicine, Khartoum, Sudan, E-mail: ammaralemam10@gmail.com; Hiba A.O. Mohamed, Nile University Faculty of Medicine, Khartoum, Sudan, E-mail: Hiba.mohamed32196@gmail.com; Elaf Sabri Khalil Mergani, Al-Neelain University Faculty of Medicine, Khartoum, Sudan, E-mail: elafsabri515@gmail.com; Aya Elshaikh Mohamedtoum Babiker, Ahfad University for Women, Faculty of Medicine, Khartoum, Sudan, E-mail: ayaelsheikh200100@gmail.com; Estbrg alsafi Mohamed ahmed, West Kordufan University, Faculty of medicine, West kordufan, Sudan, E-mail: estbrgalfeel@gmail.com; Nuha Tayseer Ibrahim Abu Dayyeh, Ahfad University for Women, Faculty of Medicine, Khartoum, Sudan, E-mail: sama.mohamed99@icloud.com.
